# Letter to the Editor: Combination of FTO and BTK inhibitors synergistically suppresses the malignancy of breast cancer cells

**DOI:** 10.7150/ijbs.132248

**Published:** 2026-04-08

**Authors:** Meichen Liu, Ziqi Zhao

**Affiliations:** 1 Department of Neurology, The First Affiliated Hospital of Dalian Medical University, Dalian, China.; 2 Department of General Surgery, The First Affiliated Hospital of Dalian Medical University, Dalian, China.

To the editor,

With considerable interest, we perused the research paper by Saad *et al.*^1^ published in the International Journal of Biological Sciences in 2025, and highly commended the significant contribution of this research in exploring the combined treatment strategy for breast cancer. This study systematically expounded the synergistic anti-tumor effect of FTO and BTK inhibitors (FB23 and Ibrutinib) in breast cancer and initially disclosed the molecular mechanism of their regulation of tumor malignant behavior through the m6A-YTHDF2-c-Myc/E2F1 axis. This work not only offers a novel combination strategy for the targeted treatment of breast cancer but also deepens our comprehension of the function of m6A modification in tumor progression. Herein, we intend to put forward several insights from the perspectives of academic and clinical translation for further investigation.

Firstly, regarding the depth of molecular mechanism interpretation, this study has shown that combination therapy inhibits tumor growth and metastasis by enhancing the m6A modification of c-Myc and E2F1 mRNA and facilitating YTHDF2-mediated mRNA degradation. Nevertheless, the direct interaction between the FTO and BTK signaling pathways and the upstream regulatory network demand further analysis. For instance, do BTK inhibitors indirectly regulate the activity or expression of FTO by influencing the activity of certain kinases? Does FTO also partake in regulating the m6A modifications of key factors in BTK-related signaling pathways, such as the BCR signaling? In-depth exploration of these issues may contribute to uncovering a more comprehensive signal spectrum of the synergistic effect of FTO-BTK and provide a foundation for optimizing the mechanism of combination therapy. Additionally, the application of FTO-BTK combination therapy in clinical practice still has a considerable distance to cover. Key considerations between combination therapy and monotherapy ought to be thoroughly discussed and taken into account, particularly regarding safety, pharmacokinetic interactions, and long-term resistance risk. Further research holds greater significance.

Secondly, in the context of clinical translation and personalized treatment, although this study demonstrated favorable synergistic effects in cell and animal models, multiple factors must be taken into account before translating it into clinical practice. Breast cancer exhibits high heterogeneity, and different molecular subtypes (such as Luminal A and B, HER2-positive, and TNBC) display significantly different responses to targeted drugs^2^-^4^. This study is primarily based on MDA-MB-231 (TNBC) and BT-549 (TNBC) cells. Whether the conclusions are applicable to HR (hormone receptor)-positive or HER2-positive breast cancer requires further subtype analysis. Additionally, the clinical research of ibrutinib in solid tumors, particularly in breast cancer, is still in its early stage. Issues such as the safety, pharmacokinetic interaction, and long-term drug resistance risk of its combined use with FB23 need to be evaluated through more preclinical and early-stage clinical trials.

In the context of the tumor immune microenvironment, recent research has indicated that BTK inhibitors possess the potential to regulate the functions of tumor-associated immune cells (e.g., B cells and macrophages)^5^-^7^, and it has also been reported that FTO can influence tumor immune escape through m^6^A modification^8^-^10^. This study primarily centers on the intrinsic behavior of tumor cells. In the future, it is feasible to further investigate the impacts of this combination therapy on immune cell infiltration, cytokine secretion, and immune checkpoint expression within the tumor microenvironment, so as to assess its potential value in immunotherapy combination strategies. It might also be of value to contemplate whether spatial transcriptomic imaging can be utilized to examine the distribution of FB23 and ibrutinib metabolites in specific organs so as to more effectively evaluate the metabolic profile of the combination therapy. Moreover, how single-cell sequencing can be employed to investigate the effects and mechanisms of the combined treatment is also a significant aspect deserving further consideration.

Finally, we highly commend the author for employing diverse techniques in mechanism research, such as RNA-seq, m6A RIP, *in vivo* transfer models, etc. These techniques offer robust support for the reliability of the conclusions. If further combined with single-cell sequencing or spatial transcriptomics, it might be possible to uncover more detailed dynamic changes in tumor cells and the microenvironment under combination therapy, thereby providing more high-resolution biomarkers for precision therapy.

We sincerely express our gratitude to the author for the systematic exploration in the crucial direction of combined targeted therapy for breast cancer. This study not only presents convincing preclinical evidence for the combined use of FTO inhibitors and BTK inhibitors but also establishes a theoretical foundation for future clinical trials. We hold the view that the principal strength of this study does not primarily reside in its exploration of more upstream molecular mechanisms. Instead, it lies in how clearly and persuasively the synergistic anti-tumor effect of the FB23 and ibrutinib combination is demonstrated and verified. Hence, beyond the reported findings, it would be of great value to contemplate whether the design and validation strategy adopted for this combination approach presents aspects that can be referenced by other researchers or further refined in future studies. It is anticipated that in future research, this combination strategy will be verified in more breast cancer models, the mechanism will be deeply explored, and there will be further breakthroughs in clinical transformation, ultimately offering new treatment options for breast cancer patients. The above viewpoints are for reference only. If there are any deficiencies, please kindly offer corrections.
